# Randomized Single-Masked Study of Preservative-Free Versus Preserved Hyaluronic Acid in Dry Eye Disease

**DOI:** 10.1167/tvst.15.11.6

**Published:** 2026-11-05

**Authors:** Radu Bucsan, Jacqueline Chua, Li Chi, Dan Deleanu, Mădălina Totir, Ruxandra Coroleucă, Alina Popa-Cherecheanu, Leopold Schmetterer

**Affiliations:** 1 Department of Ophthalmology, University of Medicine and Pharmacy Carol Davila, Bucharest, Romania; 2 Singapore Eye Research Institute, Singapore National Eye Centre, Singapore, Singapore; 3 Ophthalmology and Visual Sciences Academic Clinical Program, Duke-NUS Medical School, National University of Singapore, Singapore; 4 SERI-NTU Advanced Ocular Engineering (STANCE), Singapore, Singapore; 5 Department of Ophthalmology, Emergency University Hospital Bucharest, Bucharest, Romania; 6 School of Chemistry, Chemical Engineering and Biotechnology, Nanyang Technological University, Singapore, Singapore; 7 Centre for Medical Physics and Biomedical Engineering, Nanyang Technological University, Singapore, Singapore; 8 Department of Clinical Pharmacology, Medical University of Vienna, Vienna, Austria; 9 Fondation Ophtalmologique Adolphe De Rothschild, Paris, France; 10 Aier Eye Hospital Group, Changsha, People's Republic of China

**Keywords:** dry eye disease, hyaluronic acid, preservative-free, benzalkonium chloride, ocular surface disease index

## Abstract

**Purpose:**

To compare the effects of preservative-free hyaluronic acid (PF HA) versus benzalkonium chloride–preserved hyaluronic acid (BAK HA) on symptoms and signs of dry eye disease (DED) using identical formulations except for preservative content.

**Methods:**

In this randomized, single-masked, 6-month trial, 47 patients with DED were randomized to receive PF HA or BAK HA. The primary outcome was the change in Ocular Surface Disease Index (OSDI) score from baseline to month 6. Secondary outcomes included non-invasive tear break-up time (NIBUT), Schirmer I test, fluorescein tear break-up time (TBUT), fluorescein corneal staining (FCS), lissamine green conjunctival staining (LCS), best-corrected visual acuity (BCVA), and instillation frequency. Continuous and ordinal outcomes were analyzed using linear mixed-effects and mixed-effects ordinal logistic regression models, respectively.

**Results:**

Forty-three patients completed the study. PF HA produced a greater OSDI score reduction than BAK HA (adjusted change, −14.36 vs. −9.42; *P* = 0.001), and ∼40% of PF HA–treated patients achieved normal OSDI scores at the final visit, compared with none in the BAK HA group. At the final visit, no significant between-group differences were observed for NIBUT, Schirmer I, TBUT, BCVA, FCS, or LCS. PF HA showed more favorable longitudinal changes in symptoms and selected signs, including OSDI (*P* < 0.001), NIBUT (*P* = 0.017), FCS (*P* = 0.002), and LCS (*P* < 0.001), indicating a treatment-by-time effect. PF HA was associated with lower instillation frequency (*P* < 0.001).

**Conclusions:**

Both formulations improved DED signs and symptoms. PF HA provided greater symptomatic relief and showed more favorable longitudinal changes.

**Translational Relevance:**

These findings support the translation of experimental knowledge regarding preservative-induced ocular surface toxicity into evidence-based therapeutic decisions for long-term patient care.

## Introduction

Dry eye disease (DED) is a common, multifactorial disorder of the ocular surface.^1^^–^^3^ DED is characterized by tear film instability, hyperosmolarity, inflammation, and damage to the ocular surface epithelium.^4^ Such pathological alterations are commonly triggered by increased osmotic and inflammatory stress, which leads to elevated levels of proinflammatory cytokines and apoptotic markers at the ocular surface level.^5^^,^^6^ Clinically, DED symptoms are represented by foreign body sensation, burning, ocular dryness, and irritation, which significantly impact quality of life, visual function, and even sleep.^7^^,^^8^ The resulting socioeconomic burden on patients and healthcare systems is considerable.^9^^,^^10^

Artificial tears remain the primary therapeutic option in DED management, due to the chronic and often progressive nature of the condition, which necessitates sustained and frequent topical administration.^11^ However, the presence of preservatives in topical formulations may exacerbate ocular surface damage over time. Benzalkonium chloride (BAK), a commonly used preservative, has been associated with ocular surface toxicity, especially with prolonged use.^11^

Despite these concerns, BAK remains the most widely used preservative in topical ophthalmic formulations because of its potent broad-spectrum antimicrobial activity, low manufacturing costs, capacity to enhance corneal penetration of the active drug, and long history of safe use in multidose packaging, factors that collectively have sustained its use despite the availability of preservative-free alternatives.

Although the harmful effects of BAK have been well documented in experimental and observational studies,^12^^,^^16^^,^^19^ evidence from randomized trials comparing preservative-free versus BAK-preserved artificial tears remains limited. In addition, the differences in hyaluronic acid (HA) concentration, molecular weight, and additional excipients often confound product comparisons, making it difficult to attribute the clinical outcomes exclusively to the presence or absence of preservatives.

To address this gap, we conducted a randomized, single-masked study comparing preservative-free hyaluronic acid (PF HA) with BAK-preserved hyaluronic acid (BAK HA) in patients with DED. By using identical HA concentration formulations from the same manufacturer, we aimed to minimize the formulation-related variability and directly assess the preservative impact on both symptoms and clinical signs of DED over 6 months. This study aimed to provide clinically relevant evidence to inform treatment decisions, particularly for patients requiring long-term topical therapy. Although the adverse effects of preservatives such as BAK on the ocular surface are well recognized,^11^ few randomized controlled trials have isolated their impact by comparing preservative-free and preserved formulations of the same active ingredient, concentration, and manufacturer. Our 6-month, randomized, single-masked trial was designed to address this gap and to inform the preferential use of PF HA in the long-term management of DED.

## Methods

### Subjects

This randomized (software randomization, no stratification), single-masked, 6-month study was conducted according to the tenets of the Declaration of Helsinki and the Good Clinical Practice Guidelines of the European Union. The Ethics Committee of the Emergency University Hospital Bucharest approved the protocol (ID: 37003). The 47 patients included in the study provided written informed consent before undergoing study-related procedures at the screening visit, after explanation of the nature and possible consequences of the study. Inclusion criteria were 18 years or older at the date of the screening visit; able to understand and be willing to comply with the procedures; a history of DED (as documented by Tear Film and Ocular Surface Society Dry Eye Workshop II [TFOS DEWS II] diagnostic criteria); for longer than 3 months before the screening visit, tear break-up time (TBUT) ≤ 10 seconds and Schirmer I test ≤ 15 mm; the presence of at least one symptom of DED as burning/stinging, foreign body sensation, blurred vision, sticky feeling, sensitivity to light, itching, or pain; normal ophthalmic findings except DED; and no administration of topical lubricants 24 hours before the screening visit. Both eyes of the patients were treated and used for analysis. Any of the following excluded a patient from the study: any clinically significant slit-lamp findings at the screening visit; abnormal lid anatomy or blinking function of the eye; DED secondary to scarring; wearing contact lenses within 3 months before the screening visit or anticipating the use of contact lenses during the study; history of glaucoma; any corticosteroid treatment in the 4 weeks before the screening visit; intake of parasympathomimetic or antipsychotic drugs; topical treatment with any ophthalmic drug except topical lubricants in the 4 weeks before the screening visit; ocular surgery in the 3 months preceding the study or planned ophthalmic surgery in the next 6 months; presence of punctum plugs; Stevens–Johnson syndrome; active ocular allergies or ocular allergies expected to be active during the study period; participation in a clinical trial in the 4 weeks preceding the screening visit; or pregnancy or planned pregnancy in the next 12 months or lactating. Of the 47 enrolled patients, 43 completed the study and were included in the per-protocol analysis: 22 in the PF HA group and 21 in the BAK HA group (Table 1).

**Table 1.** Baseline Characteristics of Study Participants Stratified by PF HA and BAK HA Administration

	PF HA	BAK HA	*P* ^*^
Patients (eyes), *n*	22 (44)	21 (42)	—
Age (y), mean ± SD	49 ± 15	51 ± 15	0.567
Male gender, *n* (%)	12 (55)	4 (19)	0.036
OSDI, mean ± SD	32.33 ± 13.21	37.87 ± 14.35	0.066
BCVA, mean ± SD	0.89 ± 0.2	0.91 ± 0.19	0.706
NIBUT, mean ± SD	9.52 ± 5.38	7.92 ± 4.76	0.148
Schirmer I, mean ± SD	8.86 ± 4.32	7.36 ± 4.73	0.127
TBUT, mean ± SD	5.5 ± 2.52	5.9 ± 2.38	0.445
FCS, mean ± SD	1.05 ± 0.75	1.21 ± 0.65	0.264
LCS, mean ± SD	0.84 ± 0.83	0.71 ± 0.55	0.407
BCVA, mean ± SD	0.89 ± 0.20	0.91 ± 0.19	0.706

* *P* values for comparison of PF HA versus BAK HA at baseline using independent *t*-tests (continuous variables) and χ^2^ tests (categorical variables).

### Interventions

In the present study, we compared the effect of two types of artificial tears. To avoid possible bias induced by excipients, we selected artificial tears available in both presentations: single-dose PF and multidose BAK preserved. The two products are identical and produced by the same company (SIFI SpA, Antonio, Catania, Italy), but one of the formulations is preserved and the other is unpreserved. Lubristil with preservative (BAK HA) contains sodium hyaluronate 0.15% (the mean molecular weight of HA in both forms is in the range of 1.5–3.0 mega-Daltons [MDa]), sodium chloride, disodium phosphate dodecahydrate, sodium phosphate monobasic monohydrate, benzalkonium chloride (0.005%), and purified water. Single-dose preservative-free Lubristil (PF HA) consists of sodium hyaluronate 0.15%, sodium chloride, disodium phosphate, monobasic sodium phosphate, and distilled water. Participants were randomized into one of the groups with a pro re nata administration in both eyes for the entire study period (6 months). Patients were instructed not to administer the study treatment at least 2 hours before the study visit and to report adverse events at any time.

The study included four visits: baseline (V1), follow-up at 1 month ± 1 week (V2), follow-up at 3 months ± 1 week (V3), and follow-up at 6 months ± 1 week (V4). These time points were selected to capture early (1 month), intermediate (3 months), and long-term (6 months) treatment effects, consistent with follow-up schedules used in prior randomized trials of artificial tears in DED while limiting the burden of more frequent visits over a 6-month follow-up period.

### Clinical Evaluation

One of the most frequently used questionnaires for assessing dry eye symptoms is the Ocular Surface Disease Index (OSDI).^20^ The Asia Dry Eye Society concluded that subjective severity could indicate therapeutic efficacy in dry eye treatment.^21^ For this reason, we used OSDI as the primary endpoint of the study. Other dry eye signs such as non-invasive tear break-up time (NIBUT), Schirmer I, TBUT, fluorescein corneal staining (FCS), lissamine green conjunctival staining (LCS), best-corrected visual acuity (BCVA), and frequency of instillation, were chosen as secondary endpoints.

Clinical tests were performed for both eyes of each subject, as per the TFOS DEWS II Diagnostic Methodology subcommittee. Measurements were performed in the following order so that one did not interfere with another and to minimize their impact on tear film normal physiology: subjective assessment of dry eye symptoms (ocular comfort, OSDI); BCVA (decimal acuity where higher is equivalent to better BCVA); NIBUT; Schirmer I test; TBUT with sodium fluorescein; FCS with sodium fluorescein; LCS; and frequency of instillation (patient self-report) (Table 2).

**Table 2.** Clinical Assessment Performed at Baseline, 1 Month, 3 Months, and 6 Months

Test	Baseline	1 Month	3 Months	6 Months
OSDI^©^	+	+	+	+
BCVA	+	+	+	+
NIBUT	+	+	+	+
Schirmer I	+	+	+	+
TBUT	+	+	+	+
FCS	+	+	+	+
LCS	+	+	+	+
Frequency of instillation	−	+	+	+

BCVA, best corrected visual acuity; FCS, fluorescein corneal staining; LCS, lissamine green conjunctival staining; NIBUT, non-invasive tear film break-up time; OSDI^©^, Ocular Surface Disease Index; TBUT, tear film break-up time with sodium fluorescein.

BCVA was recorded on a 5-meter Snellen chart during each visit. OSDI was used to assess discomfort of dry eye symptoms. The questionnaire, designed for DED patients, covers three parameters: symptoms, functional limitations, and environmental triggers. A score of 0 to 12 is considered normal, a score of 13 to 22 indicates mild DED, a score of 23 to 32 indicates moderate DED, and a score of 33 to 100 indicates severe DED.^22^

NIBUT was evaluated with an Oculus Keratograph 5 M corneal topographer (Oculus Optikgeräte, Wetzlar, Germany). Patients were instructed to blink and then look straight at the camera (not directly into the light), keeping their eyes open as long as possible. The white-light camera was activated and tilted to align the camera settings icon at the level of the patient's pupil. We used the organ alignment assistant to center the patient's eye in the image. When the NIBUT measurement was activated, we received a live stream of the tear film break-up integrated into the widefield image of the patient's ocular surface. Alteration of the tear film determined irregularities of the corneal reflections of Placido rings. The time in seconds for this to happen was recorded as the NIBUT.^23^ A single NIBUT measurement was obtained per eye at each visit; measurements were not repeated or averaged.

The Schirmer I test (without anesthesia) was performed according to the TFOS DEWS II diagnostic methodology guidelines. Schirmer paper strips were inserted over the temporal one-third of the unanesthetized lower lid margin. After that, the patient was asked to close their eyes, and after 5 minutes the wetting of the Schirmer paper was measured.^20^

TBUT was measured following the guidelines mentioned above.^20^ A minimally saline-impregnated fluorescein strip was applied to the outer canthus area, and patients were asked to blink several times to spread the fluorescein over the ocular surface. Then, 2 to 3 minutes after installation, patients were asked to perform two complete blinks and then to stare straight ahead without blinking. We used 10× magnification for the slit lamp, cobalt blue light, Wratten 12 yellow filter, and a stopwatch to record the time elapsed between the second blink and the first appearance of a dry spot.^20^^,^^24^

Ocular surface staining tests (fluorescein and lissamine green) were executed per the TFOS DEWS II diagnostic methodology report.^20^ For staining severity of the ocular surface grading, we used the Oxford Scheme.^25^ Fluorescein and lissamine green paper strips were saline moisturized (excess shaken off) and applied to the outer canthus area; patients were asked to blink several times to spread the dye. The ocular surface was washed with normal saline between the tests. Fluorescein indicates cell integrity disruption (i.e., disruption in superficial cell tight junctions).^26^^,^^27^ Lissamine green is a vital dye that stains epithelial cells in case of damaged cell membranes regardless of the mucin presence.^28^^,^^29^

### Statistical Analysis

The primary endpoint was the change in OSDI over time between the two treatment groups. Secondary endpoints were changes over time in NIBUT, Schirmer I, TBUT, BCVA, and ocular surface staining (FCS, LCS), as well as frequency of instillation. Continuous baseline characteristics were summarized as mean ± standard deviation (SD) and compared between treatment groups using independent *t*-tests; categorical variables were summarized as counts and percentages and compared using χ^2^ tests.

For continuous longitudinal outcomes, linear mixed-effects models were fitted with random intercepts for participants and for eyes nested within participants to account for within-subject and within-eye correlations over time. Fixed effects included treatment group (PF HA vs. BAK HA), visit (time), and the treatment × visit interaction, with additional adjustment for sex and the baseline value of the respective outcome. Global *P* values for treatment, visit, and interaction effects were obtained using Wald tests.

Corneal and conjunctival staining scores (FCS, LCS), which follow an ordinal Oxford grading scale, were analyzed using mixed-effects ordinal logistic regression models with the same hierarchical random-effects structure (subject and eye) and the same fixed effects (treatment, visit, treatment × visit, baseline score, and sex). Associations between OSDI and NIBUT at baseline and at 6 months and between their changes over time were assessed using Spearman rank correlations. Mean bilateral NIBUT was calculated for each participant because OSDI was measured at the participant level.

The primary analysis was conducted on a per-protocol basis. A sensitivity analysis was performed using all available observations from the 47 randomized participants according to their assigned treatment group. Missing outcomes were not imputed. Mixed-effects models were fitted to account for the correlation between repeated measurements from both eyes of the same participant.

Given the absence of previous trials directly comparing identical-composition PF HA with BAK HA, the sample size was calculated based on the minimum clinically important difference (MCID) for the OSDI score (10 points). Assuming a standard deviation of 12, two-sided α = 0.05, 80% power, and 1:1 allocation, the required sample size was 40 completers. The study achieved 43 completers, thereby meeting the predefined target sample size. A two-sided *P* < 0.05 was considered statistically significant. All analyses were conducted using Stata 16 (StataCorp, College Station, TX).

## Results

Of the 47 enrolled patients, 43 completed all study visits (91.5%) and were included in the per-protocol analysis. Four patients missed the final visit and were excluded because the primary endpoint required baseline-to-V4 data. The sensitivity analysis produced findings consistent with the per-protocol analysis. At V4, the adjusted OSDI difference between the BAK HA and PF HA groups was 5.66 points (95% confidence interval [CI], 1.42–9.90; *P* = 0.009), compared with 5.24 points in the per-protocol analysis (95% CI, 0.83–9.64; *P* = 0.020). The treatment × visit interaction remained significant in both analyses (*P* = 0.016 and *P* = 0.021, respectively). Adjustment for sex attenuated the V4 treatment difference from 6.36 points (95% CI, 2.09–10.64; *P* = 0.004) to 5.24 points (95% CI, 0.83–9.64; *P* = 0.020), without changing the overall conclusion. Male sex was not significantly associated with OSDI (β = −3.51; 95% CI, −7.62 to 0.59; *P* = 0.093). The exploratory treatment × visit × sex interaction was also not statistically significant (*P* = 0.094).

Baseline characteristics were generally comparable between treatment groups (Table 1), although the PF HA group had a higher proportion of male participants (55% vs. 19%; *P* = 0.036). Baseline dry eye symptom severity (OSDI) and clinical measures (BCVA, NIBUT, Schirmer I, TBUT, FCS, and LCS) were comparable between groups (all *P* ≥ 0.066).

Both PF HA and BAK HA groups demonstrated improvements from baseline to V4 across objective and subjective dry eye parameters (Table 3). NIBUT, Schirmer I, and TBUT increased in both PF HA and BAK HA, and FCS and LCS decreased in both groups; between-group differences were not significant (all *P* > 0.05). OSDI scores improved significantly in both groups, with a greater reduction observed in the PF HA group (−14.36; 95% CI −16.25 to −12.47) compared with BAK HA (−9.42; 95% CI −11.36 to −7.49), resulting in a significant between-group difference at V4 (*P* = 0.001) (Fig. 1). BCVA showed small improvements in both groups, with no significant between-group difference (*P* = 0.136).

**Table 3.** Change in Signs and Symptoms of DED

Outcome Measurements	Baseline (Mean ± SE)	V4 (Mean ± SE)	Adjusted Change (V4–V1)	95% CI	*P* ^*^
NIBUT					0.116
PF HA	8.97 ± 0.66	11.85 ± 0.68	+2.88	1.46 to 4.30	
BAK HA	8.64 ± 0.68	13.41 ± 0.70	+4.77	3.32 to 6.22	
Schirmer I					0.705
PF HA	8.94 ± 0.75	11.71 ± 0.76	+2.78	1.75 to 3.81	
BAK HA	7.53 ± 0.76	11.29 ± 0.77	+3.76	2.71 to 4.82	
TBUT					0.203
PF HA	4.87 ± 0.93	9.90 ± 0.97	+5.03	2.74 to 7.33	
BAK HA	6.45 ± 0.95	11.68 ± 0.99	+5.24	2.89 to 7.59	
FCS					0.446
PF HA	1.10 ± 0.12	0.29 ± 0.12	−0.81	−0.99 to –0.64	
BAK HA	1.14 ± 0.12	0.43 ± 0.12	−0.71	−0.89 to –0.54	
LCS					0.888
PF HA	0.83 ± 0.09	0.02 ± 0.09	−0.81	−0.98 to –0.65	
BAK HA	0.73 ± 0.09	0.04 ± 0.10	−0.69	−0.85 to –0.52	
OSDI					**0.001**
PF HA	34.88 ± 1.11	20.52 ± 1.13	−14.36	−16.25 to –12.47	
BAK HA	35.37 ± 1.13	25.95 ± 1.16	−9.42	−11.36 to –7.49	
BCVA					0.136
PF HA	0.91 ± 0.04	1.00 ± 0.04	+0.08	0.05 to 0.11	
BAK HA	0.88 ± 0.04	0.92 ± 0.04	+0.04	0.01 to 0.07	

The *P*-values highlighted in bold are the statistically significant values.

* *P* values represent baseline-adjusted comparisons between PF HA and BAK HA at V4, derived from mixed-effects regression models with fixed effects for treatment, visit, treatment × visit interaction, sex, and baseline value. Random intercepts were specified for subjects, with eye nested within subject for eye-level outcomes.

**Figure 1.**
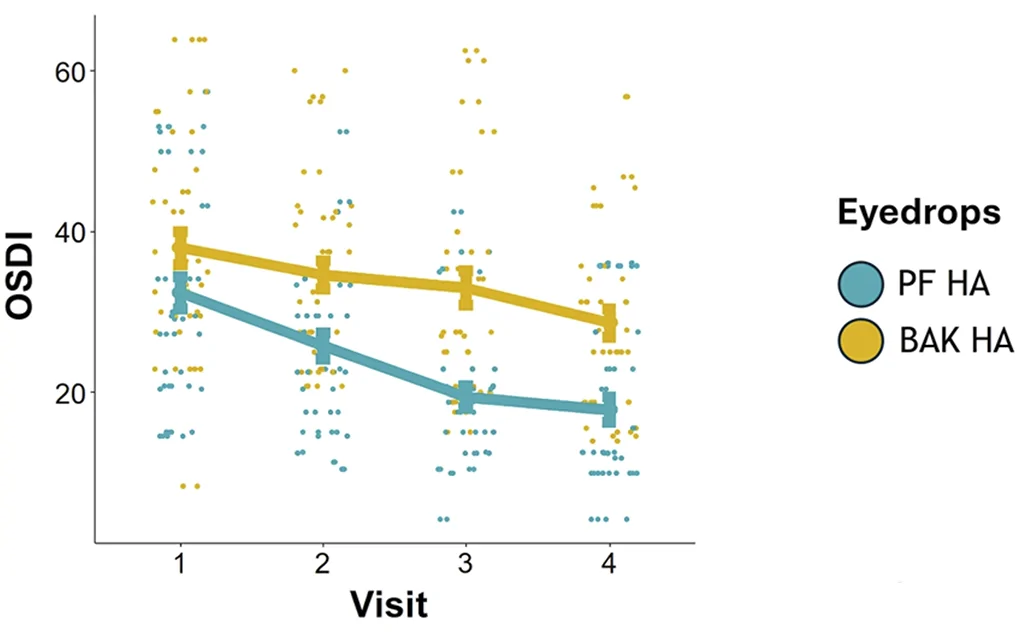
Trajectory of OSDI scores over the four study visits for PF HA and BAK HA. Both groups demonstrated progressive reductions in OSDI scores from baseline to V4, indicating symptomatic improvement. The decline was steeper in the PF HA group, particularly between V2 and V3, resulting in lower mean OSDI scores at V4 compared with the BAK HA group. Although both treatments improved symptoms over time, PF HA showed a greater magnitude of reduction.

Figures 2 and 3 show the distribution of OSDI scores at the first and fourth visits. None of the BAK HA patients achieved a normal OSDI score at V4, whereas approximately 40% of PF HA patients fell within the normal range. This reflects the significantly greater improvement in OSDI in the PF HA group compared with the BAK HA group, consistent with the mixed-effects model results (*P* = 0.001).

**Figure 2.**
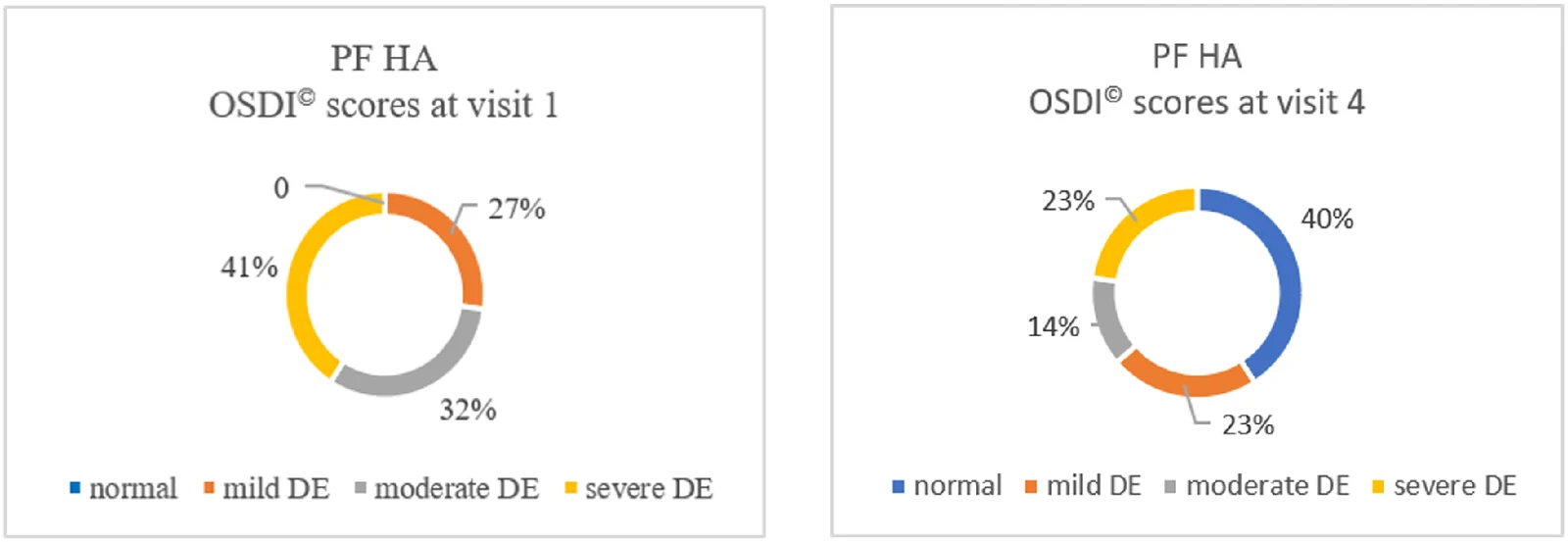
OSDI score severity at V1 and V4 for the PF HA group.

**Figure 3.**
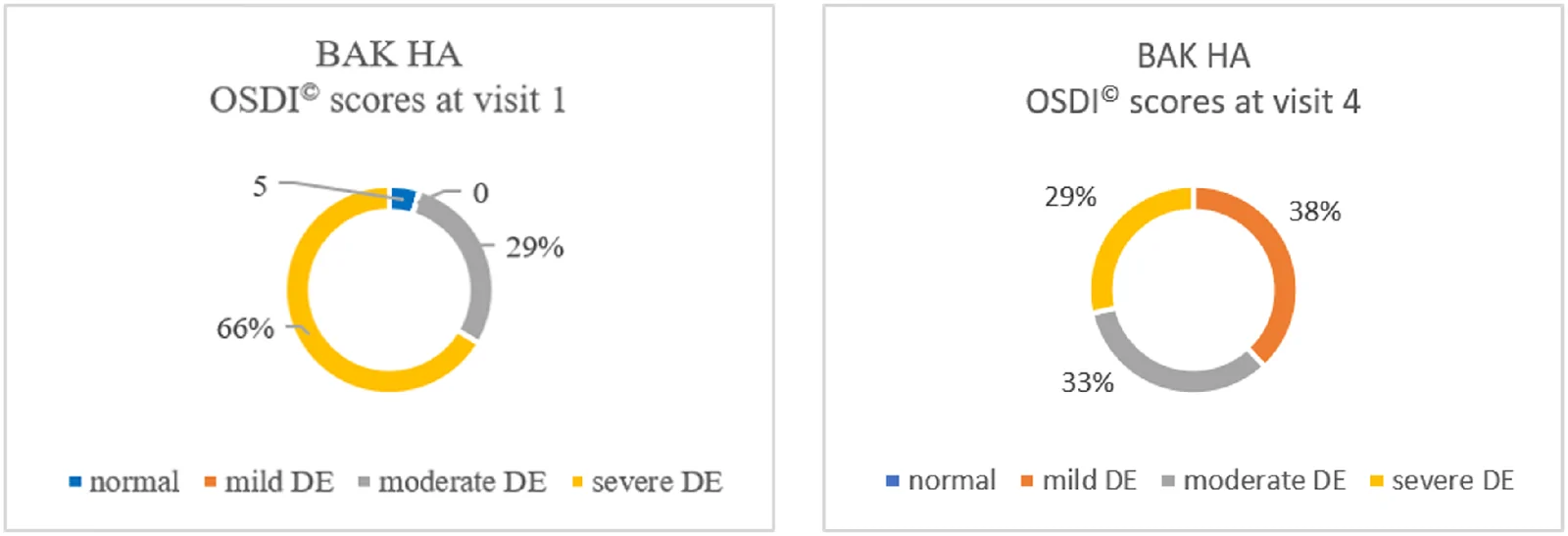
OSDI score severity at V1 and V4 for the BAK HA group.

Mixed-effects models demonstrated significant improvements over time for all dry eye measures (*P* ≤ 0.001) (Table 4). No global treatment differences were observed for NIBUT, Schirmer I, TBUT, or BCVA (*P* ≥ 0.198). A significant treatment × visit interaction was observed for OSDI (*P* < 0.001) and NIBUT (*P* = 0.017), indicating differential change over time between groups for these outcomes. No significant treatment × visit interactions were found for Schirmer I, TBUT, or BCVA (*P* ≥ 0.111), suggesting similar temporal patterns of change in these measures across treatment groups.

**Table 4.** Effects of Treatment and Time on Dry Eye Outcome

	*P* ^*^		
Outcome Measurements	Treatment (PF vs. BAK)	Visit (Time)	Interaction (Treatment × Visit)
NIBUT	0.731	**<0.001**	**0.017**
Schirmer I	0.198	**<0.001**	0.111
TBUT	0.241	**<0.001**	0.676
OSDI	0.763	**<0.001**	**<0.001**
BCVA	0.496	**<0.001**	0.203

The *P*-values highlighted in bold are the statistically significant values.

* *P* values were derived from baseline-adjusted linear mixed-effects models including fixed effects for treatment (PF HA vs. BAK HA), visit (time), and their interaction, with sex and the baseline value as covariates. Random intercepts were specified for subjects; for eye-level outcomes, an additional random intercept for eye nested within subject was included. Global *P* values were obtained using Wald tests.

OSDI and mean bilateral NIBUT were not significantly correlated at baseline (Spearman ρ = 0.058, *P* = 0.698) or at 6 months (ρ = −0.024, *P* = 0.880). Changes in OSDI and NIBUT were also not significantly correlated (ρ = −0.219, *P* = 0.159), suggesting that improvements in tear-film stability did not closely correspond with improvements in patient-reported symptoms.

Patients receiving PF HA reported fewer daily instillations than those receiving BAK HA at 1 month (3.42 ± 0.65 vs. 4.22 ± 0.85), 3 months (2.67 ± 0.87 vs. 3.91 ± 0.73), and 6 months (2.68 ± 0.84 vs. 3.52 ± 0.75) (Table 5). Mixed-effects ordinal logistic regression showed no significant difference between PF HA and BAK HA in FCS (*P* = 0.762) or LCS (*P* = 0.834) after adjusting for baseline values and sex (Table 6). However, treatment × visit interactions were significant for both FCS (*P* = 0.002) and LCS (P < 0.001), indicating that the trajectory of staining improvement over time differed between groups. During the study timeline, no adverse events were reported by the patients.

**Table 5.** Daily Eye-Drop Use by Treatment Group

Visit	PF HA, Mean ± SD	BAK HA, Mean ± SD
1 month	3.42 ± 0.65	4.22 ± 0.85
3 months	2.67 ± 0.87	3.91 ± 0.73
6 months	2.68 ± 0.84	3.52 ± 0.75

**Table 6.** Effects of Treatment and Time on Dry Eye Parameters

	*P* ^*^		
Outcome Measurement	Treatment (PF vs. BAK)	Visit (Time)	Interaction (Treatment × Visit)
Fluorescein corneal staining (Oxford Scale)	0.762	**<0.001**	**0.002**
Lissamine conjunctival staining (Oxford Scale)	0.834	**<0.001**	**<0.001**

The *P*-values highlighted in bold are the statistically significant values.

* *P* values were derived from baseline-adjusted mixed-effects ordinal logistic regression models including fixed effects for treatment, visit, and treatment × visit interaction, with sex and baseline staining as covariates, and random intercepts for subjects and eyes nested within subjects. Global *P* values were obtained using Wald tests.

## Discussion

Although BAK effects have been described previously in many trials, many formulations still contain BAK. This may be partially due to a lack of randomized controlled trials that provide the highest level of evidence. In this prospective randomized trial, we compared the effects of PF HA and BAK HA in patients with DED over 6 months. This is one of the few randomized controlled trials to control formulation differences by using identical HA concentration, molecular weight, and manufacturer in both arms, thereby isolating the effect of the preservative. We found that, although both formulations improved tear film parameters and ocular surface staining, PF HA was associated with a significantly greater reduction in patient-reported symptoms (OSDI), and a higher proportion of patients achieved normal symptom scores at the study endpoint.

Both formulations led to significant improvements in tear film parameters and ocular surface staining, but PF HA was associated with a significantly greater reduction in patient-reported symptoms, as measured by the OSDI. Notably, approximately 40% of patients receiving PF HA achieved normal OSDI values at study completion, whereas none of the BAK HA group reached this threshold. These findings indicate that both treatments are effective in improving objective measures of tear film and ocular surface health, but PF HA produced a significantly greater reduction in OSDI scores compared with BAK HA, as confirmed by baseline-adjusted mixed-effects modeling (treatment × visit interaction *P* < 0.001).

The results of this study show that, even though both artificial tears positively influenced the OSDI score, patients on PF HA recorded statistically significant lower values (*P* = 0.010), and the study population on BAK HA suffered more ocular surface disease symptoms. Although the between-group difference in OSDI improvement favored PF HA and was statistically significant (adjusted change, −14.36 vs. −9.42; a difference of approximately 4.9 points), this magnitude falls below the 10-point MCID used for the sample size calculation.

The clinical relevance of the observed effect may therefore lie less in the mean between-group difference and more in the proportion of patients reaching a normal OSDI score. This result follows the findings of two other studies^30^^,^^31^ that compared BAK-preserved artificial tears to preservative-free artificial tears. In a multicenter, prospective, randomized, open-label study by van Setten et al.,^30^ patients were followed for 8 weeks, and Nasser et al.^31^ conducted a multicenter, noninterventional, and transversal observational survey for 3 weeks. It must be pointed out here that van Setten et al.^30^ did not mention the preservative in the control group, so it can be presumed that it also included preservatives other than BAK. Kangari et al.^32^ compared 1.6-mg preservative-free hydroxypropyl methylcellulose (HPMC) artificial tears to 0.3-*g* HPMC and 0.1 mg of Dextran 70 artificial tears, BAK preserved, in a double-masked randomized clinical trial study, and no difference regarding OSDI was found after 14 days of treatment. Similarly, Safarzadeh et al.,^33^ in a randomized patient-masked clinical trial, compared two different artificial tears with similar compositions, one preservative free and the other one BAK preserved; they also found no difference regarding OSDI at 4 weeks.

Objective clinical signs, including FCS, LCS, NIBUT, TBUT, Schirmer I test, and BCVA, improved significantly over time in both treatment groups. However, no statistically significant between-group differences were observed at the final visit for these parameters. Importantly, mixed-effects analyses revealed significant treatment × visit interactions for both staining scores (FCS and LCS) and for NIBUT, indicating that the temporal pattern of improvement differed between preservative-free and BAK-preserved formulations, despite similar endpoint values. The improvement in NIBUT observed in both treatment groups, irrespective of preservative content, likely reflects a general lubricating and stabilizing effect of HA-based artificial tears on the precorneal tear film. This suggests that over a 6-month course, the mechanical and viscoelastic benefits of HA supplementation on tear film stability may outweigh any adverse influence of BAK at the concentration used in the study. However, the significant treatment × visit interaction (*P* = 0.017) indicated that the trajectory of improvement differed between formulations, with BAK HA showing a numerically greater increase at the final visit.

Both Kangari et al.^32^ and Safarzadeh et al.^33^ found no difference between arms regarding FCS, and neither did van Setten et al.^30^ On the other hand, the findings of Nasser et al.^31^ showed a significant difference between groups. The Schirmer I test results improved in both study groups with no significant difference between them. Post-intervention analysis revealed a significant reduction in LCS across both treatment groups. Our study also found no statistically significant difference between groups regarding BCVA. Kangari et al.^32^ and Safarzadeh et al.^33^ recorded similar findings. In their work, van Setten et al.^30^ found a significant difference regarding BCVA (*P* = 0.033) in favor of preservative-free artificial tears.

The dissociation observed between the greater improvement in OSDI and the absence of parallel differences in most objective signs reflects a well-recognized phenomenon in DED. Symptom severity often correlates weakly with clinical signs such as TBUT or Schirmer values. Artificial tears primarily act by enhancing ocular surface lubrication and reducing surface friction, which may in turn modulate neurosensory input and improve patient comfort without necessarily inducing proportional changes in tear production or epithelial staining detectable by standard clinical tests. However, the neurosensory mechanisms were not directly assessed in the present study, so this interpretation should therefore be regarded as a hypothesis. Our findings reinforce the importance of prioritizing validated patient-reported outcome measures, such as OSDI, as primary endpoints in DED clinical trials, in accordance with TFOS DEWS II recommendations.

PF HA users instilled drops less often, which may reflect greater symptomatic relief and could improve adherence in chronic DED management (*P* < 0.001). When van Setten et al.^30^ compared different preserved artificial tears to a preservative-free high-molecular-weight HA (0.15%), also by pro re nata administration, they found that instillation frequency was not significantly different between groups. Although the above-mentioned papers are either switch studies or compared different preserved artificial tears to preservative-free artificial tears, the strength of our study is that we used a randomized, controlled, same formulation to compare artificial tears produced by the same manufacturer.

The improvements we observed were consistent with the alleviation of patient-reported symptoms. The statistically significant difference regarding the OSDI in favor of PF HA is due to the known cytotoxic effect of BAK to ocular surface tissues.^34^ BAK can exacerbate the vicious cycle of tear film instability by inducing pro-inflammatory cytokines and oxidative stress on the ocular surface.^35^ In contrast, preservative-free artificial tears are inert and nontoxic, and they help restore tear film homeostasis without provoking inflammation, in addition to the known anti-inflammatory effect of the HA itself.^36^^,^^37^ According to the TFOS DEWS II Clinical Trial Design Report,^38^ a comprehensive approach that considers both symptoms (ocular discomfort and visual disturbance) and signs (tear film instability, ocular surface damage, ocular surface inflammation, and tear osmolarity) is of great importance in the design of DED clinical trials.

Given the long-term use of topical ocular treatments for DED, it is critical to understand how the individual components of each formulation affect the ocular surface and influence the disease progression. This is particularly significant in DED, given that the disease definition acknowledges the loss of tear film homeostasis and ocular surface inflammation as integral to the condition.^4^ In this context, it is evident that any topical treatment used for managing the condition should avoid causing undesirable side effects that could further disrupt tear film homeostasis or exacerbate ocular surface inflammation. Multidose formulations should include preservatives unless they include a mechanism for maintaining sterility of the contents, such as ABAK (Laboratoires Théa, Clermont-Ferrand, France) or COMOD (URSAPHARM, Saarbrücken, Germany).^39^ Both ABAK and COMOD rely on a one-way valve mechanism but in different ways. In ABAK, the one-way flow is enforced by the filter, which allows solution to exit but blocks microorganisms from entering during pressure equalization. In COMOD, the one-way mechanism is built into the pump system, which allows liquid to be expelled but prevents any backflow of air or fluid into the reservoir.

BAK is the most used preservative in many ophthalmic solutions, from eye drops to contact lens solutions.^34^^,^^40^^,^^41^ Although it effectively prevents microbial contamination, BAK can also adversely affect the ocular surface when used for prolonged periods. BAK toxicity on the ocular surface primarily occurs due to its detergent properties, which can disrupt the integrity of the tear film and damage the ocular tissues.^12^^–^^19^ In both tissue culture and animal models, BAK has been shown to reduce the survival of corneal^12^^,^^18^ and conjunctival^12^ cells. In animal models, BAK causes loss of conjunctival goblet cells^13^ and increases the levels of inflammatory markers in ocular tissues.^18^ Clinical manifestations of BAK toxicity include ocular surface staining,^14^^–^^16^ decreased TBUT,^14^^,^^15^ lower Schirmer scores,^15^ worse OSDI scores,^14^^,^^16^^,^^17^ subconjunctival inflammation,^19^ and ocular surface disease symptomatology.^15^ Several studies on glaucomatous^42^^–^^46^ and some on dry eye^30^^,^^31^ populations demonstrated that preservative-free formulations can improve signs and symptoms compared to BAK-containing drops. Most of these are switch studies, with the original BAK preserved formulations being switched to PF formulations. In contrast, our study was a randomized trial, comparing the PF HA and BAK HA arms from the start.

Improvements in NIBUT were not significantly associated with improvements in OSDI, indicating that changes in tear-film stability did not closely parallel changes in patient-reported symptoms. This finding is consistent with the recognized discordance between symptoms and clinical signs in DED.^47^^–^^49^ However, the lack of correlation should be interpreted cautiously, given the relatively small sample and the variability inherent in tear-film measurements.

For our groups, the significantly greater OSDI improvement observed in the PF group compared to the BAK group represents an absence of BAK-induced discomfort. On the other hand, significant improvement in both groups represents a result of the protective effect of artificial tears on the ocular surface. The pro re nata administration allows dosing tailored to fluctuate patient needs, reflecting the real clinical use. Fixed-dose administration ensures stable therapeutic levels, but they carry a higher risk of cumulative adverse effects, especially if symptoms are not constant or if a patient’s need for the medication fluctuates. Patients included in the present study kept a personal diary on administered dose, and no maximum number of administrations was imposed.

### Novelty and Contribution

Previous studies have examined the ocular surface effects of BAK, principally in switch-design or glaucoma medication studies, but our trial contributes new evidence in three respects. First, it is among a small number of randomized controlled trials to compare preservative-free and BAK-preserved artificial tears using otherwise identical HA formulations from a single manufacturer, thereby isolating the effect of the preservative from confounding differences in HA concentration, molecular weight, or excipients. Second, it evaluated a pro re nata dosing regimen over a 6-month period, reflecting real-world use rather than a fixed-dose, short-term switch protocol. Third, it combined a validated patient-reported outcome (OSDI) with a comprehensive panel of TFOS DEWS II–recommended objective signs, allowing direct comparison of the relative sensitivity of symptom- and sign-based endpoints to preservative exposure. These design features distinguish our study from prior switch-based or short-duration comparisons and support its relevance for long-term, real-world DED management.

### Clinical Implications

Our findings provide randomized, formulation-specific evidence supporting the preferential use of preservative-free artificial tears for long term DED management, although confirmation in larger, multicenter, double-masked trials would strengthen these conclusions. Adopting preservative-free formulations could optimize symptom control, minimize ocular surface toxicity, and support sustained treatment adherence, with particular relevance for patients requiring chronic, frequent dosing.

### Study Strengths and Limitations

The strengths of our study include its randomized design, extended follow-up period, and the use of HA formulations with identical concentration and composition from the same manufacturer, minimizing variability related to active ingredients. Treatment was administered on a pro re nata basis, resulting in variable exposure between participants and treatment groups. Furthermore, dosing frequency may have been influenced by symptom severity, and greater exposure could itself have affected subsequent outcomes. This bidirectional relationship limits causal interpretation of the association between formulation and treatment response. Our study was limited by the inability to implement a double-masked design, as the packaging differences between the preservative-free and BAK-preserved products prevented complete masking of both participants and investigators. Although efforts were made to minimize bias through standardized assessments and single-masked outcome evaluation, the absence of double-masking may have introduced some degree of observer or participant bias. Attrition was low and balanced between groups, but four participants were eliminated who did not complete the final visit, which may have introduced attrition bias. However, the sensitivity analysis using all available data produced similar findings. Sex distribution was not balanced between groups. Although the analysis was adjusted for sex, the small number of men, particularly in the BAK HA group, limited our ability to determine whether treatment effects differed by sex.^50^ Another study limitation is represented by the lack of tear osmolarity or inflammatory mediator assessment, which does not adequately explore the biological reasons for why the well-known BAK-induced discomfort occurs.^40^ Our study aim was a clinical exploration, not identification, of pathophysiological mechanisms involved in ocular damage. Further studies incorporating molecular and imaging biomarkers may further elucidate the pathways through which preservatives influence ocular surface homeostasis.

## Conclusions

This randomized study, designed following TFOS DEWS II recommendations for diagnosis, evaluation, and clinical trial methodology, compared PF HA in single-dose form with BAK-preserved HA in multidose packaging. Our findings indicate that PF HA provided greater improvement in dry eye symptoms, together with a more favorable trajectory of change in select objective signs, compared with BAK-preserved HA. These results support the preferential use of preservative-free formulations in the long-term management of patients with DED to optimize both clinical outcomes and patient-reported symptom relief.
